# Sense of psychological ownership in co‐design processes: A case study

**DOI:** 10.1111/hex.13886

**Published:** 2023-10-27

**Authors:** Anette Juel, Lene L. Berring, Annette Erlangsen, Erik R. Larsen, Niels Buus

**Affiliations:** ^1^ Psychiatric Research Unit Psychiatry Region Zealand Slagelse Denmark; ^2^ Mental Health Centre Copenhagen Danish Research Institute for Suicide Prevention Hellerup Denmark; ^3^ Department of Regional Health Research University of Southern Denmark Odense Denmark; ^4^ Department of Mental Health Johns Hopkins Bloomberg School of Public Health Baltimore Maryland USA; ^5^ Center of Mental Health Research The Australian National University Canberra Australian Capital Territory Australia; ^6^ Copenhagen Research Center for Mental Health Mental Health Centre Copenhagen Hellerup Denmark; ^7^ Translational Neuropsychiatry Unit, Department of Clinical Medicine Aarhus University Aarhus Nu Denmark; ^8^ School of Nursing and Midwifery, Faculty of Medicine, Nursing and Health Monash University Clayton Victoria Australia

**Keywords:** case study, ownership, participatory research, service users, website design, workshops

## Abstract

**Introduction:**

Service users are increasingly participating in health research. Although collaborative research is assumed to give users a sense of psychological ownership, little is known about the specific psychosocial processes through which ownership develops and is displayed. The present study yields insight into a process in which service users, researchers and a website designer collaborated to design a website.

**Aim:**

The aim of this study was to explore how participants developed and displayed feelings of ownership during a collaborative process to design a website.

**Methods:**

A case study design was adopted by which audio recordings were subjected to thematic analysis and interpreted by drawing on the concept of psychological ownership.

**Findings:**

A sense of psychological ownership of the website design process emerged in two distinct and overlapping phases. In the first phase, ‘sense of ownership during the early design phase’, only researchers and the website designer displayed a sense of ownership, which was facilitated by the research context preceding the collaborative workshops. In the second phase, ‘sense of ownership during the collaborative design phase’, service users gradually started to develop parallel feelings of ownership that were facilitated by workshop design activities. These activities enabled service users to increasingly control the process, to invest themselves in the process and to gain intimate knowledge of the process and its outcome. Service users' sense of ownership was displayed in their statements about the website and its elements.

**Conclusion:**

Participants engaged in codesign processes may develop a sense of psychological ownership at different speeds because of contextual factors. It is important to take this into account as it may complicate the formation of egalitarian work groups.

**Patient and Public Contribution:**

Parents of children with suicidal behaviour and a counsellor participated as service users in a website design process.

## 1 INTRODUCTION

Contemporary healthcare policies increasingly encourage service user participation in the planning and development of healthcare services^1^ and in healthcare research.^2^ One approach to service user participation is experience‐based codesign, which enables people with lived experiences to collaborate with professionals to codesign more beneficial healthcare services.^3^ Furthermore, service users' participation in research may improve research outputs by, for example, informing the choice of research priorities and disseminating findings.^4^ In the context of this paper, we understand the term participation as an active partnership between researchers and service users in the research process.^2^ There is a paucity of research into actual participatory research processes, which will be the focus of the current paper.

Conceptual models, such as Arnstein's^5^ ladder of citizen participation, have been used to describe service user participation in research. In these models, different levels of participation are displayed, each step up reflecting service users' increasing participation and empowerment.^6^ Consultation is considered the lowest level of participation; here, service users are asked about their views, although their opinions do not necessarily inform research decisions. Collaboration is considered an intermediate level of participation in which service users and researchers collaborate closely together in an on‐going partnership. Lastly, user‐controlled participation reflects the highest level of service user influence; here, users are in charge of all decisions regarding the design and conduct of a research project. Yet, such models have been criticised for being too narrowly focused on decision‐making power while failing to consider the impact of other factors related to the collaborative process. Service user participation is typically a dynamic process and may consist of different levels of participation.^7^

Service users who participate in collaborative or user‐controlled research practices are assumed to feel ownership of the research,^6^, ^8^ but little evidence exists to support this claim. Salsberg et al.^9^ focused on ownership in a study where researchers collaborated with community stakeholders to develop an intervention to increase physical activity among children in elementary schools. The study sought to identify actions and strategies that would result in a shift of ownership and decision‐making from researchers to stakeholders. The authors reported that different aspects of the collaborative process prompted this shift of ownership, for instance, the leadership taken by a researcher at the beginning of the process, the participation of a core group of people, individual personalities who worked well together and project objectives that were well aligned with stakeholders' professional roles. However, it remains unclear how these aspects conveyed a sense of ownership, and the study failed to provide a theoretical understanding of ownership. An analysis without such conceptualising risks becoming unfocused and having limited explanatory value.^10^

Psychological ownership may be defined as a mental state in which individuals feel as if a material or immaterial object is theirs. It contains a cognitive dimension as individuals are aware that they perceive an object as theirs and express this through words such as ‘mine’ or ‘ours’ and an affective dimension as individuals feel emotionally attached to this object and may express this through positive statements about the object. Psychological ownership differs from legal ownership; whereas the rights associated with legal ownership are specified and protected by society, the rights of psychological ownership are defined by the individuals.^11^, ^12^

To perceive psychological ownership, three motivations must be in place.^12^ First, individuals must perceive that they are able to manipulate and control the object, which enhances their feelings of being effective and competent. Second, they must be able to see a meaning in the object because it hereby becomes a symbol of their self‐identity and of high social value. Third, they must be able to feel at home with the object; they are familiar with it and it provides a kind of personal security. A feeling of psychological ownership may arise through three distinct and complementary paths. This feeling emerges when individuals (1) exercise control over the object, (2) gain intimate knowledge about the object and (3) invest themselves in the object, that is, their thoughts and emotions, and see this investment reflected in the object. Contextual factors influence heavily if a feeling of psychological ownership arises. These factors may be structural, that is, rules, norms and hierarchy, or cultural, that is, traditions and beliefs in a society.^11^, ^12^ Thus, scientific investigations of ownership should take such contextual factors into consideration.

In 2017, a group of Danish researchers obtained financial support to develop a psychoeducational website that would support parents in coping with the difficulties that arise in the wake of their children's suicidal behaviour. We chose to develop a website because information and support would be easily accessible and scalable. Service users who, in the context of this paper, were parents of children with suicidal behaviour and a counsellor were invited to participate in the design of this website through a series of workshops. It was crucial to investigate ownership in collaborative practices as ownership constitutes as a significant evaluation outcome and influences the degree to which service users disseminate the results of the research process.^13^ Drawing on the aforementioned conceptualisation of psychological ownership, the aim of the present study was to explore how workshop participants developed and displayed feelings of ownership during the collaborative process of designing a website.

## 2 METHODS

### 2.1 Design

A case study design was adopted^14^ for its usefulness in gaining an understanding of the mechanisms that are at play in a particular event, such as a collaborative design of a website. The study drew on two types of data: the primary data were the audio‐recorded workshops, while the secondary data consisted of observations made by the first author, who was an observing participant in the workshops. Besides having an educational background as a registered nurse, the first author was a PhD‐trained qualitative researcher with a particular interest in group interactions.

### 2.2 Workshop participants

The workshop participants (see Table 1) were service users, researchers (including the authors A. J., L. L. B., A. E. and N. B.) and a website designer. Service users were parents who self‐identified as having a child at risk of suicidal death or injury, and a counsellor who provided psychoeducation for parents of children with suicidal behaviour. Although the counsellor had originally intended to only provide support for the parents, she became equally involved in the design process. In recruiting parents, an information leaflet that outlined the project was distributed to venues that were possibly in contact with parents. The leaflet also provided contact details of the first author, who handled the enrolment process. One parent offered her participation after reading about the project; three parents were recruited through centres for child and adolescent psychiatry, one parent were recruited through a nongovernmental organisation and two parents were recruited through snowballing.

**Table 1:** Workshop participant characteristics.

Workshop participant	Position of authority	Aspects of knowledge
Service user no. 1	Mother of a daughter with SB	9 years of lived experience.
Service user no. 2	Mother of a daughter with SB	2 years of lived experience.
Service user no. 3	Mother of a daughter with SB	14 years of lived experiences.
Service user no. 4	Mother of a son with SB	12 years of lived experience.
Service user no. 5	Mother of a daughter with SB	18 years of lived experience.
Service user no. 6	Mother of a daughter with SB	2 years of lived experience.
Service user no. 7	Mother of a son with SB	2 years of lived experience.
Service user no. 8	Counsellor at an NGO	9 years of experience of supporting parents.
Researcher no. 1	Professor	20 years of experience with qualitative and participatory research and research on suicide prevention.
Researcher no. 2	Associate professor	20 years of experience with research on suicide prevention.
Researcher no. 3	Associate professor	18 years of experience with qualitative and participatory research.
Researcher no. 4 (the first author)	PhD student	7 years of experience with qualitative research.
Website designer	Software designer	10 years of experience with software programming.

Abbreviations: NGO, nongovernmental organisation; SB, suicidal behaviour.

All participants were assumed to possess different kinds of knowledge, which legitimised their participation in the workshops. Service users had experiential knowledge of the challenges that parents face when a child is suicidal and contributed first‐hand knowledge of valuable support. Two parents stated that they were living together with their child, while five parents reported that their child was living in a care facility. Moreover, three parents had upper secondary education, while four parents had postsecondary education. Researchers had theoretical knowledge about suicide prevention, research methods and procedures. The website designer had knowledge about software programming.

The workshops were conceptualised through a slow, open format, which meant that participants could change gradually over time. Eight service users participated in the first workshop, but two parents concluded their participation shortly thereafter. An additional three parents were invited to participate but never attended the workshops. Whereas all researchers participated in the first workshop, only the first author and the website designer participated in all workshops.

### 2.3 Workshops

In applying a participatory approach to the website design, we followed general guidelines for service user participation.^2^, ^15^, ^16^, ^17^, ^18^, ^19^, ^20^ Eight workshops were organised by the first author over the course of a 2‐year period, starting in May 2019 and ending in March 2021 (see Table 2). The first five workshops were held in person in a public meeting venue in Copenhagen and, due to Covid‐19 restrictions, the subsequent three workshops were held as online sessions. Each workshop had a planned duration of 3 h.

**Table 2:** Workshop characteristics.

Workshop number#, month and year	Physical/online	Workshop participants	Duration
#1, May 2019	In person	Service user no. 1–8	2 h, 59 min
		Researcher no. 1–4	
		Website designer	
#2, June 2019	In person	Service user no. 2, 3, 5, 6, 8	2 h, 44 min
		Researcher no. 4	
		Website designer	
#3, August 2019	In person	Service user no. 1–3, 6, 8	2 h, 46 min
		Researcher no. 1, 4	
		Website designer	
#4, December 2019	In person	Service user no. 2, 3, 5, 6, 8	2 h, 47 min
		Researcher no. 4	
		Website designer	
#5, August 2020	In person	Service user no. 1–3, 5, 6, 8	3 h, 1 min
		Researcher no. 4	
		Website designer	
#6, January 2021	Online	Service user no. 2, 3, 5, 6	2 h, 8 min
		Researcher no. 1, 2, 4	
		Website designer	
#7, February 2021	Online	Service user no. 2, 3, 5, 6, 8	2 h, 50 min
		Researcher no. 4	
		Website designer	
#8, March 2021	Online	Service user no. 2, 3, 5, 6, 8	2 h, 3 min
		Researcher no. 4	
		Website designer	

The first author and the website designer prepared the workshop agendas. The first workshop was less structured; after a brief introduction to all participants, a general discussion of wishes and expectations to the website evolved. All subsequent workshops had a more structured agenda and listed various topics related to the website design. The agenda was sent to all participants by email ahead of each workshop. The first item on the agenda at the beginning of each workshop was reflections and experiences since the last meeting and the closing item was reflections on the discussions that had evolved during the workshop. The first author and the website designer, who led the meeting, introduced the points on the agenda and explained how they would like service users to contribute with suggestions to the outline of topics and design for the website. After each workshop, a resume was sent to all participants. The resume briefly stated key points raised and decisions made during the meeting. In a few instances, service users were invited via email to comment on specific issues in the intervals between workshops. Service users received travel reimbursement and an honorarium payment after each attended workshop. The audio recordings of the workshops were transcribed verbatim by a research assistant. Subsequently, the first author checked the accuracy of these transcriptions against the recordings. Observational data were mainly focused on the psychosocial atmosphere during the workshops, for example, if the first author observed interactional patterns in the participants' formal and informal discussions. Therefore, the workshop transcripts constituted the main data in the analysis.

Different formats of psychoeducation were suggested for the website, including (1) video clips that recaptured parents' experiences of providing care for children with suicidal behaviour, (2) questions and answers (Q&A), addressing specific topics of relevance, (3) a chat robot, in the form of an avatar, that is, a computer animated adult female, who, in segmented video clips combined through programming, asked parents questions and provided advice on issues of concern and (4) virtual peer support groups.

### 2.4 Ethics

The Danish Data Protection Agency approved the present study ahead of the workshops (REG‐049‐2018, Region Zealand). All workshop participants who consented to participate were given written and verbal information about the study ahead of their participation. An anonymised version of the workshop transcripts was used for the analysis. Although the counsellor repeatedly offered her support to parents over the course of the workshops, none of the parents made use of this offer.

### 2.5 Data analysis

The first author conducted the analysis and developed the interpretation aided by the last author. Data were organised, analysed and interpreted using a thematic approach.^21^ The transcripts were carefully read to allow the author to familiarise herself with its content, after which an open coding was conducted. During the coding process, codes that summarised and rephrased data segments were developed. Memos summarising and exploring thematic content and initial ideas of analytical interest were drafted while coding. The subsequent steps regarding the organisation of the analysis were discussed with the last author. Codes related to website elements were categorised in separate documents under the following topics: the chat robot, video clips, Q&A, virtual peer group and website layout. These were then reread, focusing on how each website element had been negotiated over time and which participants had contributed to this negotiation. Subsequently, the codes were grouped and summarised according to three questions: (1) *How did the descriptions of the website element and its perceived relevance develop over time?*, (2) *How did the decision to adopt the website element develop over time?*, and (3) *How did participants work with this website element over time?* By comparisons across website elements, we sought patterns, congruencies and divergences while considering possible ways to explain these preliminary analytical insights.

The concept of psychological ownership^11^, ^12^ was then chosen to further progress the data analysis and interpretation.^22^ We explored specific data segments that indicated participants' sense of ownership. This could be statements or behaviours that displayed that participants cognitively perceived the website design process as theirs or that participants were emotionally attached to the process. By drawing on our preliminary analytical insights, we explored how the three distinct paths facilitated a sense of psychological ownership including how contextual conditions, negotiations and adopted working practices had influenced the emergence of felt ownership.

## 3 FINDINGS

**Figure 1:**
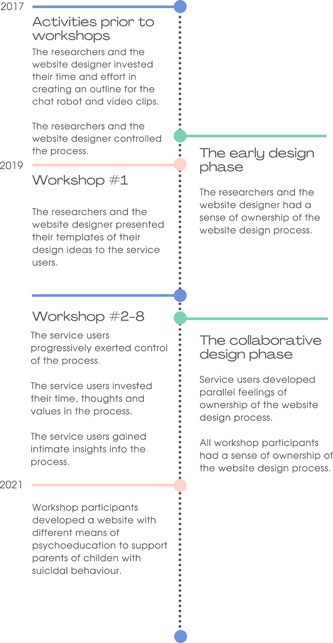
Sense of ownership during the website design process.

Over the course of eight workshops, participants collaborated to design a website with psychoeducation for parents of children with suicidal behaviour. The participants developed a sense of psychological ownership of the website design process during two distinct and overlapping phases. The first phase, ‘sense of ownership during the early design phase’, comprised a prolonged period before the workshops and during the first workshop. In this phase, only the researchers and the website designer displayed a sense of ownership, which was facilitated by the research context preceding the collaborative sessions. The second phase, ‘sense of ownership during the collaborative phase’, started to emerge at the second workshop. In this phase, service users gradually began to develop parallel feelings of ownership, encouraged by the working practices adopted for designing the website. Here, the website design turned into a collaborative effort. Each of the two phases is described below with illustrative quotations. Figure 1 provides an illustrative overview of the interpretation.

### 3.1 Sense of ownership during the early design phase

In the period leading up to the first workshop, the researchers and the website designer had compiled a proposal with their ideas for the website. These ideas comprised a chat robot and video clips capturing experiences of parents providing care for children with suicidal behaviour. Moreover, the researchers had obtained funding to develop a website with these elements. This contextual condition positioned them in control of the process and afforded them the power to define the preliminary procedures for service user participation. They decided on the number of workshop participants and invited service users. They arranged the workshops, defined the structure and agenda of each workshop and decided how they would like service users to contribute to the group work. They developed templates of their ideas for the website, which they presented to the service users during the first workshop. Hence, they had invested a considerable amount of time and effort in sketching a path for the workshops and in creating an outline for the chat robot and video clips. Owing to these early design activities, the researchers and the website designer perceived psychological ownership of the website process before initiating the workshops.

Their sense of ownership was displayed in their voiced enthusiasm about their design ideas. For instance, when introducing the chat robot, researcher no. 2 stated: ‘We thought it might be a helpful tool, well it is our idea, so now you are welcome to tell us if it is totally off’. The researcher used the expression ‘our idea’ and emphasised its potential value, thereby suggesting that the researchers and the website designer would be reluctant to abandon this idea. As such, the website design context positioned the service users with limited control over this part of the process. They were, at the beginning of the workshops, unable to make decisions because they lacked insight into research processes and had limited knowledge of software programming, research methods and potential tools for psychoeducation.

Although the researchers and the website designer had initiated the design process ahead of the workshops, they rhetorically tried to downgrade the significance of this, possibly to support a sense of a genuine collaborative partnership. This occurred at workshop no. 1 when they informed the service users that they had the power to make decisions regarding all website content and refrained from mentioning that the chat robot and video clips were mandatory elements because they had been specified in the funding proposal. When the researchers and the website designer presented their arguments about the value of the chat robot and video clips, service users rejected these by suggesting that Q&A and virtual peer support groups would be more helpful. This rejection suggested that service users did not feel ownership over these website elements. The researchers and the website designer did not challenge the service users' stance but promptly accepted their suggestion to develop a Q&A section, possibly to mediate their reluctance towards the chat robot and video clips.

The following data extract illustrates how service users challenged the researchers' arguments for producing the video clips and instead argued in favour of creating virtual peer support groups. The extracted dialogue occurred during workshop no. 1, where one researcher had asked the service users about their wishes and expectations to the website:

Service user no. 4: ‘The thing that provides the most support is to be in direct dialogue with someone who has been through the same experience’.

Researcher no. 1: ‘I think parents need to know that their behaviour is normal given the context of this disaster’.

Service user no. 8: ‘But that would require that they meet someone who can tell them that’.

Researcher no. 1: ‘That's what I'm hoping the video clips can instigate [addresses service user #7 who is shaking her head]… you stress me out when you shake your head at me like that’.

Service user no. 8: ‘I think it's about meeting others face to face in groups, to enable that reflection in others, and I just can't see that happening right now [on the website], but I think it would be great if you made a virtual peer support group where parents can meet on the internet, I'm sure it's technically possible’.

Service user no. 1: ‘It is a good message to have on a website, that there is a special room that can strengthen the capacity to endure’.

Researcher no. 2: ‘Well, I am open to all suggestions, but I think that to make a virtual peer group where parents can meet on this website and talk would require monitoring by a professional who can intervene, and that would be very expensive’.

The data extract illustrates how a service user suggested developing a virtual peer support group on the website, which was endorsed by other service users. One of the researchers immediately challenged this suggestion, arguing that video clips would provide a similar type of support to parents as peer groups. Although service users voiced considerable scepticism and continued to stress the relevance of virtual peer support groups, the suggestion was eventually dismissed by a researcher. The data extract exemplifies how researchers were reluctant to dismiss the video clip idea and how, due to their context‐generated position of power, they allowed themselves to reject the suggestion proposed by service users.

Similarly, the researchers and the website designer would occasionally disregard knowledge shared by service users if it conflicted with their own knowledge about website design and research methods. This occurred, among others, in workshop no. 3. Here, a researcher introduced a list of questions to be used in the interviews with parents for the video clips. Service users suggested omitting the final question: ‘Why do you think this happened to you and your family?’ because they were concerned that it might leave the interviewees with feelings of distress and guilt. However, the researchers ignored their concern and replied that this specific issue was addressed several times during the interview and therefore needed to be explored also at the end of the interview. To address their concerns, it was decided that a debriefing session would be conducted at the end of the interviews.

Although these examples illustrate that some of the service user input was ignored, the researchers and the website designer continued to emphasise that the lived experiences and opinions of the service users were essential for constructing a useful website. Unlike the researchers and the website designer, service users continually needed to legitimise their participation in the workshops by sharing their experiences, values and opinions, while realising that their potential contributions could be dismissed. This led to an initial distrust in the researchers and the website designer, which seemed to prevent the service users from feeling psychological ownership.

### 3.2 Sense of ownership during the collaborative design phase

Through the collaborative design activities, which were applied to create the website, gradual feelings of ownership started emerging among service users. Thus, in this second phase, all participants perceived psychological ownership of the design process. The activities gave service users increasing control of the process and allowed them to invest themselves in the process and to gain intimate knowledge of the process. We observed their growing sense of ownership in the ways in which they gradually changed their statements about the website and its elements and in their displays of agency in the workshops.

In the second phase, service users seemed to become more confident as to their role in the design process while accepting the decisions that were beyond their control. At this stage, the design process was more equally shared and controlled among all workshop participants. Service users exerted a greater influence on website design; for instance, they decided the wordings for the questions in the Q&A. Moreover, although they had been unable to overturn the decision regarding the video clips, they decided (1) which topics should be addressed in the video clips, (2) how the video clips should be recorded and (3) how the video clips would be organised on the website. In a similar vein, they decided which topics should be addressed by the chat robot, how it should appear on the website, how its voice should be and its name. In this way, the content of the website gradually began to represent items that were rooted in the service users' wishes and priorities. Moreover, a collaborative partnership developed as service users increasingly challenged and changed the group's activities to fit with their preferences. This observed behaviour demonstrates their increasing control of the process, which gave rise to their sense of ownership.

Service users also invested themselves in the design process. This investment was, among others, witnessed through the amount of time spent on the project, but also through their sharing of private thoughts, emotions and values. Service users openly shared the most difficult challenges that they had faced as parents to underpin their suggestions for video clip topics. Similarly, they revealed their emotional reactions to researcher‐produced questions included in the chat robot as arguments for why they thought questions should be formulated differently. They also shared both positive and negative experiences of accessing other websites to underpin their website layout preferences. This process implied that the website gradually started to reflect their experiences and values. This investment was increasingly reciprocated by the researchers and the website designer, who shared personal anecdotes about their own lives. As such, the various personal investments gradually formed a collectively shared practice.

A third factor that helped facilitate a growing sense of psychological ownership was the intimate insights that service users gradually gained into the web design process. Over the course of the 2‐year period, service users became familiar with the other workshop participants and the design activities. They learned that their personal investments were typically welcomed by the other participants, who responded with supportive and encouraging remarks. Thus, the workshops became a safe place where they could share their knowledge and rapport was established between the workshop participants. This growing familiarity enhanced service users' commitment, which was also underscored by their continued attendance at the workshops. Moreover, service users felt responsible for the website. An example of this was clear from workshop no. 5. Here, one of the researchers invited the service users to describe their thoughts regarding their participation. One service user who had participated in all workshops but refrained from responding to emails in between workshops said: ‘I am not sure that I am the right person to be here, you know, do I contribute enough… I have not always felt that I give enough, considering that I said yes to be here’ (Service user no. 6). In this data extract, the service user voiced uncertainty concerning whether her contributions were fulfilling, seemingly taking on responsibility for creating a useful website.

This state of psychological ownership was displayed in the way that service users gradually changed their thoughts about the chat robot and the video clips. For instance, they initially voiced their preferences for a Q&A section and virtual peer support groups rather than video clips. However, at workshop no. 5, when the researcher showed a pilot video clip, two service users immediately provided their positive feedback:

Service user no. 3: ‘I showed my husband the video clip that you sent, and he was deeply moved by it, he felt really bad for that mother [in the clip], I think it worked really well that you asked a question [at the beginning of the clip]’.

Service user no. 2: ‘I agree, it [the interview question] provides context so you know where you are’.

The service users found that the video clip was authentic and it produced an emotional reaction in viewers. As they were in charge of setting up for the video clips, they decided to include interview questions as part of the video clips and in this way, they voiced their opinion about the questions, saying that they set the context for the parents' unfolding narrative.

Similarly, service users gradually changed their view of the chat robot. At first, their response was negative. They thought it would possibly generate feelings of loneliness because it was unable to listen and to respond in a suitable manner and that it would possibly feel like a provocation. After investing time in designing the chat robot and gaining more influence on its appearance, they gradually changed their opinions:

Service user no. 2: ‘I think that she can actually calm you down when you're stressed out’.

Service user no. 8: ‘I agree’.

Service user no. 1: ‘Yes, it provides something else than reading, it conveys a human feeling even if it's just a robot’.

Service user no. 2: ‘There is something therapeutic about her, she provides some sort of comfort’.

Service user no. 1: ‘We can talk about this, we can verbalise this, it's amazing what it can do’.

From initially being opposed to the idea of the chat robot, service users started thinking that it would potentially serve to relieve distress and help verbalise difficult topics, seemingly comparing the robot to a therapist. The positive statements about the video clips and chat robot demonstrate how service users gradually assumed ownership of these website features. These statements were observed in workshop no. 5, which suggests that an amount of time was necessary for ownership to emerge. Similar to the researchers and the website designer, service users now spoke of the website as theirs.

## 4 DISCUSSION

In this case study, two distinct phases were identified of how workshop participants developed and displayed feelings of psychological ownership of a collaborative website design process. In the context of this study, service users were parents of children with suicidal behaviour and a counsellor. Initially, only the researchers and the website designer had such feelings, which arose through their preparations ahead of the workshops. In the second phase, a parallel feeling of ownership emerged among service users through their participation in design activities, which took place during workshops. In the final workshops, all participants were committed to the process of designing the website.

The literature is characterised by a paucity of studies on ownership in participatory research. Salsberg et al.^9^ stated that their adopted engagement strategies had enabled community stakeholders to take/feel ownership for the research process and resulting intervention. They reported, among others, that having a stable group of stakeholders aided the ownership process.^9^ However, their assumption that these strategies produced stakeholder ownership seems oversimplified as it was not supported by theory or empirical evidence.^9^ The present study showed that participation in collaborative design activities gradually facilitated a sense of ownership among service users. Different factors related to the website design process enabled this feeling to arise, for instance, their increasing control of and personal investments into the process. Guided by the theory of psychological ownership, we were able to identify data documenting their sense of ownership through positive statements about the website and its elements. Nevertheless, we agree with Salsberg et al.^9^ that having a core group of participants is beneficial for the advancement of the process. Although our workshops had a slow, open format, they changed minimally over time and a core group of service users eventually attended most workshops. This implied that rapport was gradually established between workshop participants, which may have enhanced service users' commitment even more.

To improve future collaborative practices, it is important to reflect on how power dynamics affect participatory research. In the field explored herein, awareness of power dynamics is particularly important as service user participation risks becoming a way of legitimising already‐taken decisions.^23^ The researchers' authority in the website development space afforded them legitimate power over nonresearchers, which may be seen as challenging the equity among collaborative partnership members.^24^ Even though the researchers and the website designer approached the website design process with the best of intentions, aiming to share power in the decision‐making processes, they had, in fact, made some key decisions before initiating the codesign process that they imposed on the group's work. Storming and norming processes are normal when establishing effective work groups,^25^ and these processes were further complicated by a heightened awareness of power imbalance between the workshop participants. Power and collaborative relationships were negotiated in the group processes and, in time, decision‐making power was effectively shared. However, we ultimately do not know if the observed group processes would have been less intensive without the researchers' authority and prework exercise of power, and we caution against over‐interpreting normal group processes in codesign as being contingent on equity in decision‐making.

Although shared decision‐making is important, Tritter and McCallum^7^ argued that delegating power to service users does not alone guarantee successful participation. Success is equally dependent on adjusting the collaborative practices to fit the service users' capabilities and desires.^7^ In the present study, different types of participation were used; for instance, when the researchers and the website designer asked service users for feedback on the chat robot and video clips, this could be considered consultation. Yet, when service users decided to include a Q&A element on the website and developed a set of questions, this resembled collaboration. The combination of participation levels, in the form of consultation and collaboration, was well suited to the parents who participated as service users in this study. The parents prioritised a considerable deal of their available time to ensure their child's well‐being^26^, ^27^; thus, it would not have been feasible for them to commit to the workload required to developing, for example, a chat robot. However, service users could have participated earlier in the process, for example, contributing to the research proposal as recommended by Manafo et al.^28^ Such a measure might have diminished the authority of the researchers and the website designer and ensured a more even power balance between participants. Furthermore, we suggest that our collaborative practices were successful because service users came to feel ownership over the process and showed continued commitment to the workshops.

### 4.1 Strengths and limitations

As noted by Tritter and McCallum,^7^ participatory research should involve a multiplicity of different stakeholders. Despite elaborate recruitment efforts, stakeholders in the present study only comprised of mothers of children with suicidal behaviour and a counsellor. It would have been preferable to also involve fathers. However, fathers may be more reluctant to participate, by being less willing to share personal experiences than mothers^29^ and to seek psychological support in general.^30^ Consequently, it was not possible to draw any conclusions as to whether feelings of ownership may vary by gender. They differed with respect to years of lived experience, suggesting that service users had different insights regarding the care pathway and, thus, brought different perspectives and preferences to the design process. After the first workshop, two service users withdrew their participation. We did not document their reasons for dropout, but the workshops may not have been perceived as a safe setting by those who opted out of the study after their suggestion to develop virtual peer support groups was dismissed.

The analysis included data from workshops conducted over an extended period of time, which allowed us to map the negotiation of ideas and design activities over time. Thus, as a data collection method, audio recordings and observations of workshops proved to be a powerful tool with which to study collaborative processes.^22^ The approach was, however, less suitable for gaining an understanding of psychological states, such as sense of ownership. For this reason, our findings regarding ownership were limited to recorded statements about the website and its elements and did not apply to ownership per se. Moreover, it was not possible to elaborate on all elements of the concept from the data; for instance, we were unable to explore how the design context and activities affected the three underlying motivations. Follow‐up interviews would be an efficient way to further deepen and strengthen the interpretation of data. It is also important to note that the first author held a ‘participant as observer’ position^31^ as she participated in the workshops and subsequently led the analysis and interpretation of data. While participation may lead to a problematic ‘closeness’ to the situation, the opportunity to collaboratively study the audio recordings at a later time facilitated a ‘distance’ to the situation, which assisted in developing a balanced and nuanced interpretation. Our interpretations were strongly influenced by the selection of the theoretical framework, and to ensure that the interpretations were fruitful and firmly grounded in the data, we closely reread the workshop transcripts during data analysis. Finally, we used peer debriefing and kept an audit trail via memos,^32^ which contributed to enhance the trustworthiness of the interpretations.

## 5 CONCLUSION

Although participatory research is assumed to provide stakeholders with a sense of ownership, little is known about the specific psychosocial processes in which an ownership feeling develops and is displayed. The present study identified two phases in which feelings of ownership emerged in workshop participants engaged in a collaborative process to design a website. In the first phase, only the researchers and the website designer demonstrated these feelings facilitated by their design activities ahead of the workshops. In the second phase, service users gradually displayed parallel feelings of ownership encouraged by the collaborative design activities, which took place during the workshops. The participatory process characterised by psychological ownership is one in which service users progressively exert control, invest their time, thoughts and values and acquire intimate insights into the process, fostering a close connection with the other participants and commitment to the outcomes. Moreover, our findings suggest that the early design activities amplified the power imbalance between participants, which further challenged the development of ownership among service users. The present findings provide novel insights into the under‐researched field of ownership and suggest ways of codesigning healthcare interventions that are likely to foster a sense of ownership. Although our findings are particular to our research context, future research may investigate and further elaborate on how collaborative research partnerships influence sense of ownership, for instance, if all group members start participating in the process at the same time or have a foundational understanding of the dynamics of collaborative research processes.

## AUTHOR CONTRIBUTIONS

**Anette Juel**: Conceptualisation; methodology; resources; formal analysis; investigation; writing—original draft preparation; writing—review and editing; project administration; funding acquisition. **Lene L. Berring**: Conceptualisation; methodology; resources; project administration; funding acquisition; writing—review and editing. **Annette Erlangsen**: Conceptualisation; resources, project administration, writing—review and editing. **Erik R. Larsen**: Resources; writing—review and editing. **Niels Buus**: Conceptualisation; methodology; formal analysis; writing—review and editing; project administration; funding acquisition.

## CONFLICT OF INTEREST STATEMENT

The authors declare no conflict of interest.

## Data Availability

The data are not publicly available due to privacy or ethical restrictions
